# Public Emotional and Coping Responses to the COVID-19 Infodemic: A Review and Recommendations

**DOI:** 10.3389/fpsyt.2021.755938

**Published:** 2021-12-14

**Authors:** Weijun Ying, Cecilia Cheng

**Affiliations:** ^1^School of Education, Johns Hopkins University, Baltimore, MD, United States; ^2^Department of Psychology, The University of Hong Kong, Pokfulam, Hong Kong SAR, China

**Keywords:** anxiety, eHealth, pandemic, false information, fake news, health literacy, misinformation, coronavirus

## Abstract

Since its onset in early 2020, the coronavirus disease 2019 (COVID-19) pandemic has adversely affected not only the physical but also the mental health of people worldwide. Healthcare professionals and laypersons have sought to learn more about this novel and highly transmissible disease to better understand its etiology, treatment, and prevention. However, information overload and misinformation related to COVID-19 have elicited considerable public anxiety and created additional health threats. Collectively, these problems have been recognized by the World Health Organization as an “infodemic.” This review provides an overview of the global challenges posed by the COVID-19 infodemic, and used the psychological entropy model as a guiding framework to explicate the potential causes of the infodemic and identify potential solutions to mitigate impacts on public health. We first examine the role of anxiety in information processing and then delineate the adverse impacts of the infodemic. Finally, we propose strategies to combat the infodemic at the public, community, and individual levels.

## Introduction

The coronavirus disease (COVID-19) outbreak was declared a pandemic by the World Health Organization (WHO) in March 2020 (1). Although a prominent concern is that the causal virus—SARS-CoV-2—may cause lethal damage to the respiratory system, scholars have also warned of the mental impacts of the COVID-19 pandemic on people living in affected regions [e.g., (2, 3)]. Depression and anxiety are the primary mental manifestations experienced worldwide during the COVID-19 pandemic [e.g., (4, 5)]. Many of these mental disturbances are related to the COVID-19 “infodemic,” a portmanteau of “information” and “epidemic” coined by the World Health Organization (6) to describe the overabundance of information and misinformation disseminated during the COVID-19 pandemic. Research on the mental effects of the pandemic has shown that the COVID-19 infodemic is an “invisible disaster” with serious and far-reaching deleterious impacts (7, 41). In this review, we examine mental health issues related to the COVID-19 pandemic and investigate coping responses used by the general public to deal with these effects. Specifically, we give an account of the plethora of false or misleading information widely disseminated *via* offline or online media during the pandemic and provide recommendations for tackling these timely issues.

## Coping With Uncertainty and Anxiety Aroused by the COVID-19 Pandemic

The widespread uncertainty and panic that have arisen during the pandemic are attributable in part to the novelty of COVID-19, the etiology and treatment of which were unknown during the initial stages of the outbreak. According to the psychological entropy model (8), uncertainty can be modeled as entropy in one's mental state, and anxiety arises when perceived uncertainty increases. The model explicates how thoughts and feelings of uncertainty are intensified and how people attempt to mitigate such heightened levels of perceived uncertainty. The model highlights two major types of control: pragmatic and epistemic control (9). The former refers to the undertaking of immediate actions to reduce or terminate a perceived threat, and the latter refers to the active gathering of information regarding the nature, cause, and future development of the perceived threat.

The psychological entropy model is applicable to the COVID-19 pandemic because many people have been motivated to reduce their perceived uncertainty to a manageable level. In addition to passively following the COVID-19 preventive measures issued by governments and health authorities [e.g., the United States Centers for Disease Control and Prevention (CDC), WHO], one common way to enhance pragmatic and epistemic control is to search for information in an attempt to instantly mitigate the heightened anxiety, threat, and fear elicited by the pandemic. In today's digital era, the sheer volume of information flow can be emotionally taxing for information consumers (10).

## Information Seeking as a Coping Strategy and Associated Problems

The overabundance of information and processing issues have been recognized by the WHO as the core of the “infodemic” phenomenon. In the present digital age, many people deploy information seeking as a coping strategy to mitigate heightened anxiety and gain pragmatic and epistemic control over perceived health threats and uncertainty during the COVID-19 pandemic. Such information-seeking tendencies have been exacerbated by the stay-at-home orders and restrictions implemented in many countries, such as lockdowns, curfews, and teleworking (11). People also reported expending more effort and time than usual on seeking information through the Internet when living under these measures for disease prevention (12). Although information seeking can mitigate uncertainty and anxiety, this coping strategy can also elicit psychological problems if information seekers are exposed to false and inaccurate information (i.e., misinformation), overwhelmed by information (i.e., information overload), or both.

With respect to information overload, the social media monitoring team of the Vaccine Confidence Project showed that the term “COVID-19” was mentioned more than 690 million times in digital and social media messages globally between January and May 2020 (13). As ~4.75 million COVID-19-related messages are disseminated daily, it is impossible for Internet users to read all these messages, and thus they can only selectively view a portion of the information posted on websites. During the initial phase of the pandemic, many Internet users reported feeling confused and overwhelmed by the abundance of information available online (14).

With respect to misinformation, about half of respondents in a UK survey mentioned that they had browsed fake or inaccurate news online (15). People have posted countless pieces of false and inaccurate COVID-19 information online due to a lack of thorough understanding of the novel disease, including its etiology, transmission mode, and effective treatments (15, 16). This problem has been exacerbated by the dissemination of false and inaccurate information by renowned authoritative figures. For instance, the French Minister of Health, Olivier Véran, shared inaccurate information stating that the use of anti-inflammatory drugs (e.g., ibuprofen and cortisone) might worsen the infection. Owing to the minister's social status and reputation, some French citizens warned their social network members not to take anti-inflammatory drugs. Even if such information is not entirely false, it may not be equally applicable to every circumstance or individual (16). In a press briefing in April 2020, the US President Donald Trump suggested inaccurately that disinfectants could be used to treat COVID-19. Accidental poisoning cases related to disinfectants and bleach were found to increase in the month after the press briefing [(17), May].

During the pandemic, many people have expressed difficulties in distinguishing between true and false COVID-19 information (15). Conflicting information disseminated by government bodies and public health organizations (e.g., the CDC and WHO) has further perplexed the public that has created considerable social mistrust and complicated individuals' decision-making (13). Although some governments have communicated positive and accurate preventive information *via* telehealth to relieve the public's mental health burden during the pandemic (18), some people do not believe or are unable to digest information from official sources. Meanwhile, others continue to obtain false information from dubious online sources, despite the availability of accurate preventive information from reliable sources (19).

The COVID-19 infodemic poses considerable threats to both physical and mental health, A number of social issues have emerged, such as public fear, stigma, and discrimination toward those who are or may be infected by the disease (20). The unprecedented volume of inaccurate or false information on the disease has caused massive confusion among the general public, and has increased uncertainty and anxiety. Ironically, attempts to gain epistemic control *via* information seeking during an infodemic do not mitigate heightened anxiety levels and may even aggravate mental health problems (20).

When facing an infodemic, people may expend excessive time and energy over-interpreting information, and thus they may fail to react in a constructive manner (20). Under involuntary quarantine, loneliness and weariness are typical immediate emotional reactions. These strong unpleasant feelings can even result in undesirable consequences. One man in India committed suicide after being hospitalized for COVID-19 with an uncertain outcome [(21), May]. Another person from India committed suicide soon after learning of his infection due to worries of infecting other villagers (22). These cases demonstrate that despite having some knowledge of COVID-19, the sources of information and how it is processed can be problematic (20).

The COVID-19 infodemic has exacerbated emotional problems among the public. A recent survey in China showed that the prevalence of depression and anxiety increased due to frequent exposure to social media (23). Another Chinese study indicated that over-exposure to pandemic-related media was a predictor of acute stress (24). Similar findings were derived from a study conducted with adults in the UK and the US (14). Specifically, respondents who more frequently sought COVID-19-related information on the Internet tended to experience higher levels of anxiety regarding COVID-19 infection. Heightened anxiety levels among this group were also related to compromised sleep quality.

The studies discussed above indicate that the COVID-19 infodemic has far-reaching consequences for mental health. Although mitigation measures have been largely effective in protecting people from COVID-19 infection and limiting the spread of this highly transmissible disease, they have also created considerable hurdles to accessing mental health services, social support, and social contacts, particularly for people in low and middle socioeconomic groups (25). Such hurdles may increase people's tendency to turn to the Internet for information. The extensive dissemination of false and misleading messages regarding the novel disease and precautionary measures has created additional challenges (26). For instance, many sources have falsely stated that boiling water, snake oil, silver, and burning incense can be used to treat COVID-19 [(27), March]. Online messages urging people to hoard protective face masks have led to unreasonable price hikes (28). Some conspiracy theorists have described COVID-19 as a bioweapon and claimed that 5G technology enables the spread of the virus [(29), January]. When COVID-19 vaccines were first rolled out, a number of conspiracy theories have also emerged, claiming that the newly developed vaccines cause COVID-19 variants, that the government put microchips in vaccines to track citizens, and that vaccines can rewrite DNA [(30), June]. These fabricated messages and conspiracy theories have intensified vaccination anxiety and hesitancy, and have reduced vaccination intention (31).

COVID-19 misinformation has prompted the public to adopt inappropriate precautionary measures and misled health professionals to prescribe treatments that deviate from the scientifically approved usage and targets of medications. For instance, hydroxychloroquine—a drug with antimicrobial immunomodulatory properties—has been wrongly touted as a cure or prophylactic for COVID-19. Some doctors prescribed the medication during the early stages of the COVID-19 pandemic because of the limited pool of available data (32). As a result, a sudden surge in demand for hydroxychloroquine has disrupted the normal supply chain and severely deprived patients who use this medication for its approved purposes.

The infodemic continues to grow, despite considerable progress in preventing and treating COVID-19 and battling the infodemic. New sources of fake, false, and inaccurate information continuously emerge in huge volumes. Although a number of vaccines have been developed and there is growing empirical evidence demonstrates their efficacy, sources continue to disseminate false claims regarding vaccine lethality and side effects. Such false claims discourage some people from accepting COVID-19 vaccination (33). One of the most widely disseminated false claims is that vaccines can affect fertility. Since December 2020, many women have refused the COVID-19 vaccine due to their heightened fears of infertility. However, the British Fertility Society has clarified that there are no theoretical grounds or empirical evidence to show that any of the vaccines influence the fertility of women or men [(34), March].

## Proposed Approaches to Combat the COVID-19 Infodemic

Given the numerous impacts of the infodemic on physical and mental health, developing effective methods to protect people from these effects is necessary. Scholars have estimated that the disease outbreak severity can be reduced by decreasing the amount of online misinformation by 10% or encouraging at least 20% of the population not to share fake or unverified information (35). To help combat the infodemic, we propose measures to be carried out at the public, community, and individual levels.

At the public level, social media can serve as a useful tool to tackle the infodemic, despite also being the primary route for spreading misinformation (36). Social media can contribute to controlling the infodemic in three main ways. First, public health organizations can use social media to prevent or minimize the spread of fake news and raise public awareness by disseminating reliable information and actively communicating with target groups in the community. Second, social media can serve as a tool for public health surveillance. For instance, governments can collaborate with social media companies to utilize big data analytics to unveil emerging health trends, track behavioral changes, and predict potential outbreaks. Third, social media can serve as an educational tool. Governments and public health organizations can help curb the spread of false information by teaching people how to critically evaluate the credibility and reliability of such information and encouraging them to stop sharing messages that contain questionable or unverified information.

Conflicting information provided by government bodies and public health organizations can be confusing to the public (13). Thus, governments and public health organizations should work together to develop communications regarding the prevention of COVID-19, taking advantage of the popularity of social media to disseminate consistent and reliable information. The information should be uploaded to credible websites to provide a reliable source of trustworthy information and build public trust.

Governments and social media companies are recommended to use technology-based measures to detect and control rumors. Specifically, with the advancement of artificial intelligence technology, machine learning can be used to identify misinformation on the Internet. Novel machine-learning models, such as deep neural networks, have great potential to propel the invention and development of rumor detection methods (37). Greater effort and resources should be devoted to the development of more effective automated programs for combatting the infodemic.

“Risk communication” methods have been recommended for fighting the infodemic at the community level (35). Risk communication can bridge the gap between what experts expect people to know and what people want to know. The purpose of risk communication is to clarify uncertainties and establish trust within the community. Three key elements are crucial for achieving this goal. First, people are advised to honestly and clearly engage in self-evaluation of what they know and do not know. Second, governments and public health organizations should monitor and attend to the public anxiety that often emerges within communities during disease outbreaks. Third, governments and public health organizations should debunk myths and rumors in a proactive and timely manner to curb their viral spread in the community.

At the individual level, eHealth literacy should be cultivated among the public to facilitate the identification of reliable information sources (38). eHealth literacy and trust in reliable sources can mitigate the heightened psychological distress elicited by the infodemic and reduce the maladaptive tendency to avoid information on preventive measures. Specifically, information consumers should be made aware that it is normal to feel distressed or anxious after processing health information, but such unpleasant emotions can function to increase one's motivation to comply with preventive measures for health maintenance (38). Information consumers who experience distress and anxiety are easily lured into believing false information that they believe is useful (38). The likelihood of adopting effective preventive measures depends largely on whether one trusts the health recommendations given by authorities. In this light, government bodies and public health organizations should collaborate to gain public trust and ensure that people voluntarily adopt the recommended preventive measures in a timely manner. Individuals who deliberately avoid information about preventive measures are less likely to comply with such measures (38). In this case, avoidance is a maladaptive strategy for coping with the infodemic. In the short term, avoidance helps individuals to avoid the expected anxiety that they associate with actively searching for COVID-19-related information (14). In the long run, individuals with eHealth literacy and access to trustworthy information sources who actively search for information will be more equipped to manage their uncertainty and foster pleasant emotions by gaining pragmatic and epistemic control.

In addition to eHealth literacy, general critical health literacy is essential for mitigating the adverse effects associated with the infodemic (39). In particular, public health authorities should formulate policies to help cultivate public health literacy, such as maintaining trustworthy health information sources that are convenient to access and easy to understand. Combating the infodemic requires a joint effort from public health authorities and the public. If government bodies and public health authorities take responsibility for proactively creating trustworthy information sources and building health literacy among the public, information consumers may choose to trust the health information provided by these authorities. As a result, the adverse impacts of the infodemic can be minimized.

The recommended measures for combating the infodemic among the general public are equally applicable to healthcare professionals. Findings have indicated that healthcare professionals tend to perform no better than laypersons in detecting false health-related news or reports (40). Healthcare professionals are advised to be critical when making health decisions, be sensitive to the credibility of information sources, and perform fact-checks when confronted with information disseminated through questionable sources (12).

In conclusion, the present review seeks to raise public awareness of the COVID-19 infodemic, highlight its adverse impacts, and offer recommendations for addressing these problems at the public, community, and individual levels. During the COVID-19 pandemic, the infodemic has had an immense detrimental impact on the physical and mental health of people worldwide. Although the infodemic is a novel phenomenon, its present and imminent adverse consequences should not be ignored, and immediate action is needed. Governments, public health organizations, and the public should cooperate to combat the COVID-19 infodemic.

## Author Contributions

WY and CC contributed to literature review and wrote the first draft of the manuscript. CC obtained funding and supervised the project. All authors have reviewed and approved the final version of the manuscript for publication.

## Funding

This project was funded by General Research Fund (Project Number: 17400714) of the Government of the Hong Kong Special Administrative Region and the Seed Fund for Basic Research (Project Number: 201711159216) of the University of Hong Kong. The funders had no role in manuscript writing or the decision to submit the paper for publication.

## Conflict of Interest

The authors declare that the research was conducted in the absence of any commercial or financial relationships that could be construed as a potential conflict of interest.

## Publisher's Note

All claims expressed in this article are solely those of the authors and do not necessarily represent those of their affiliated organizations, or those of the publisher, the editors and the reviewers. Any product that may be evaluated in this article, or claim that may be made by its manufacturer, is not guaranteed or endorsed by the publisher.
